# Research on High Performance Milling of Engineering Ceramics from the Perspective of Cutting Variables Setting

**DOI:** 10.3390/ma12010122

**Published:** 2019-01-02

**Authors:** Rong Bian, Wenzheng Ding, Shuqing Liu, Ning He

**Affiliations:** 1 Industrial Center, Nanjing Institute of Technology; Nanjing 211167, China; dwz198151@njit.edu.cn (W.D.); liushuqing@126.com (S.L.); 2 Jiangsu Key Laboratory of Precision and Micro-Manufacturing Technology, Nanjing University of Aeronautics & Astronautics; Nanjing 210016, China; drnhe@nuaa.edu.cn

**Keywords:** milling, ceramics, ductile machining, PCD, corner radius, material removal rate

## Abstract

The setting of cutting variables for precision milling of ceramics is important to both the machined surface quality and material removal rate (MRR). This work specifically aims at the performance of corner radius PCD (polycrystalline diamond) end mill in precision milling of zirconia ceramics with relatively big cutting parameters. The characteristics of the cutting zone in precision milling ceramics with corner radius end mill are analyzed. The relationships between the maximum uncut chip thickness (*h*_max_) and the milling parameters including feed per tooth (*f*_z_), axial depth of cut (*a*_p_) and tool corner radius (*r*_ε_) are discussed. Precision milling experiments with exploratory milling parameters that cause uncut chip thickness larger than the critical value were carried out. The material removal mechanism was also analyzed. According to the results, it is advisable to increase *f*_z_ appropriately during precision milling ZrO_2_ ceramics with corner radius end mill. There is still a chance to obtain ductile processed surface, as long as the brittle failure area is controlled within a certain range. The appropriate increasing of *a*_p_, not only can prevent the brittle damage from affecting the machined surface, but also could increase the MRR. The milling force increases with increasing MRR, but the surface roughness can still be stabilized within a certain range.

## 1. Introduction

### 1.1. Machining of Ceramics

Thanks to the favourable combination of outstanding mechanical, thermal and chemical properties, advanced engineering ceramics, such as oxides, carbides, nitrides, and borides, have received growing attention in modern industries. It has broad application prospects in mechanical, aerospace, automotive, electrical, chemical and biological engineering etc. For example, the transplantation of artificial joints requires that the alternative materials have stable chemical properties in the living body, good biocompatibility, no component element dissolution and no stimulation to the body, and zirconia and alumina ceramics are ideal choices. The Power-MEMS (MEMS based power sources) project developed by researchers has a turbine diameter of only 20 mm, a working speed of at least 500,000 rpm, and a high temperature of about 1200 K. This requires a small material density, high temperature resistance, and high strength. Comprehensive assessment, advanced ceramic composite (SiN-TiN) can meet the demand [1,2,3,4]. As an example, zirconia ceramics (ZrO_2_) have the highest fracture toughness (*K*_IC_ between 4 and 12 MPa/m^1/2^) and flexural strength, combined with ionic conductivity, excellent thermal insulating properties and biocompatibility properties. Unique properties make it very suitable for advanced applications and some precision components, such as pump impellers for turbomachinery, diesel injection micro-nozzles, micro-fluidic devices, micro-molds, dental and orthopedic implants. It is also used for electronic product housing material. The case of smart phones and watches is also increasingly using zirconia ceramics with a smooth surface and good wear resistance [3]. 

Like some typical difficult-to-cut materials such as titanium alloys and high-temperature alloys, keeping high machined surface quality and improving machining efficiency has always been a key issue. Research works on tool wear, surface integrity and vibration are very necessary for understanding and optimizing the machining process [5,6,7,8,9]. Cutting force models are also used for estimation of the cutting forces to avoid unfavourable cutting parameters that lead to wrong use and premature failure of the tools [10]. At present, the machining of engineering ceramics also faces similar problems, especially high surface quality is required. Damage-free machining of ceramics has been studied by several researchers in recent years. It is reported that ceramic materials can be machined in ductile regime by decreasing the undeformed chip thickness to a sufficiently small value called “critical depth of cut” or “critical chip thickness”, *d*_c_, to get a very smooth machined surface without brittle damage [11,12,13]. Most of the research works are focused on precision or ultra-precision grinding of engineering ceramics in ductile or partial ductile mode [14,15]. However, precision grinding is time consuming, and it has certain limitations when it comes to complex three-dimensional structures. 

To meet the demand of fabricating complex structures, alternative processes to machine ceramics have been also studied in the last decades. For example, Ferraris et al. [16,17] investigated the machining behavior of many electrically conductive ceramics, including Al_2_O_3_-, ZrO_2_-, and Si_3_Ni_4_-based ceramic composites via electrical discharge machining (EDM). The results demonstrated the feasible manufacturing of complex shapes and micro features. However, the approach suitability is mostly limited to some electrically conductive ceramics, such as carbides and composites. Furthermore, it is the reported the machined surface presents thermal-induced micro-cracks, which may cause adverse effects on the operational performance of some precision components. With the development of 3D printing technology, research on the production of ceramic parts through additive manufacturing (AM) technology has emerged [18,19,20]. The AM processes for ceramics use ceramics in powder form, and AM techniques can directly be applied to a ceramic slurry and provide in-situ sintering. By this way, three-dimensional complex parts can be easily obtained. However, depending on the final required tolerance and surface quality of the part, especially for the precision part, final machining still might be needed [3].

### 1.2. Ductile Mode Milling with Ultra-Hard Tools

Micro milling has many advantages, such as high machining flexibility, removing materials quantitatively, and machining components with complex three-dimensional structures [21,22,23]. With the development of ultra-hard materials, different kinds of ultra-hard cutting tools such as diamond coated tools, polycrystalline diamond (PCD) tools, and cubic boron nitride (CBN) tools have been used. This makes it possible that materials with high hardness or brittleness, such as engineering ceramics [24,25], tungsten carbide [26,27], silicon [28] and pure tungsten [29] etc., can be directly machined in ductile mode by hard milling. Unlike pure cutting, in which the relative tool sharpness, known as RTS (the ratio of the uncut chip thickness to the cutting edge radius) should be ≥ 10. For some forming gear tools, RTS can be accepted ≥6. In the case of ductile mode milling, RTS is always below the critical value, which promotes the formation of large compressive stress into the chip formation zone, and enhances the plastic deformation of the undergoing material. Successful attempts on ductile mode milling have been conducted recently. Matsumura and Ono [30] reported that grooves with axial depth of cut in the range of 15~20 μm can be machined in glass if the CBN ball end mill is tilted at a certain angle in the feed direction. Bian [24] investigated the feasibility of ductile mode milling of ZrO_2_ ceramic with diamond coated end mills, and presented a mirror-like machined surface and a three-demensional structure. Cheng [31] studied the process of micro milling tungsten carbide with PCD tools, achieved nanometric surface finish and micro-rib with a width-depth ratio of 1:10. Wu [27] also studied the tool wear of self-developed PCD tool in micro milling of tungsten carbide, and discussed the effect of tool wear on cutting force, surface quality and machined groove shape. 

Thus, ductile mode milling seems a feasible way to achieve complex shape and crack free surface for hard and brittle materials, such as engineering ceramics. It is also an effective complement to the following finishing of EDM ceramics and 3D printed ceramics precision parts. However, there is still a noticeable lack of experience in this specific topic, especially in precision milling of ceramics in hard state. For instance, serious tool wear despite using ultra-hard tools and very low materials removal rate due to the micro level cutting parameters [24,32,33,34].

This work specifically aims at the performance of PCD end mills with corner radius in precision milling of zirconia ceramics in the hard state with Vickers hardness about HV 1180. The relationship between cutting parameters and tool corner radius are analyzed. High performance milling experiments with exploratory cutting parameters that cause uncut chip thickness larger than the critical value have been carried out to help understand the characteristics of machined surface formation. Based on that, the author put forward the strategy to improve the material removal rate while ensuring a high machined surface quality when precision milling ZrO_2_ ceramics.

## 2. Analysis on Variables Setting in Precision Milling of Ceramics

### 2.1. Characteristics of Parameters in Precision Milling Ceramics

In the precision milling process, cutting parameters such as feed per tooth (*f*_z_), axial depth of cut (*a*_p_), are normally set to a very small value. When the parameters are reduced to a certain extent, the geometric characteristics of the cutting zone will change. The actual uncut chip thickness *h* is not the same as that in conventional milling.

**Figure 1:**
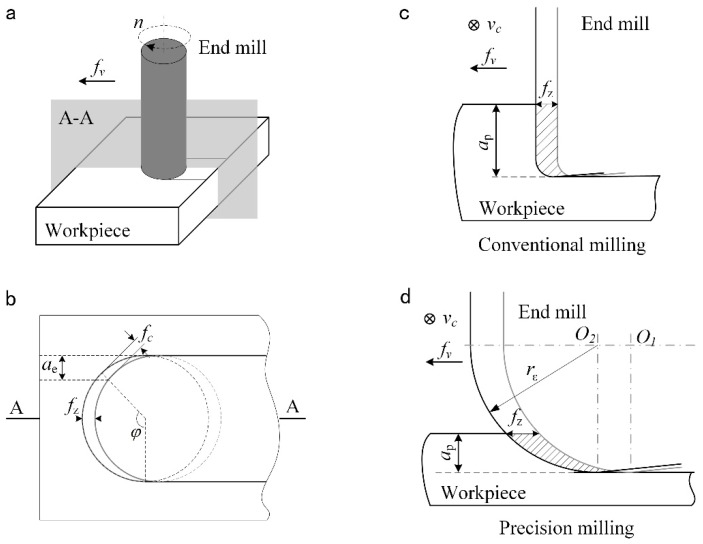
Schematic of groove milling from (**a**) side view, (**b**) top view and zoom in of cutting zone in (**c**) conventional milling and (**d**) micro precision milling.

**Figure 2:**
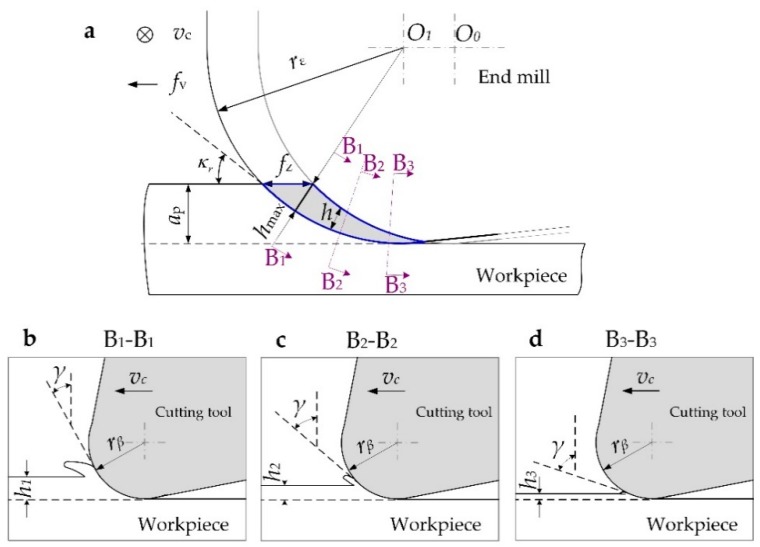
Schematic of (**a**) cutting zone in precision milling and the cross-section view of the uncut chip thickness at different position of (**b**) B1, (**c**) B2 and (**d**) B3.

As shown in Figure 1, when groove milling with a flat-bottom end mill, the radial uncut chip thickness *f*_c_ at any position during the single cutting process of the cutting edge changes with the tool rotation angle *φ*, (*f*_c_ = *f*_z_ sin*φ*). In the feed direction (A-A section, *φ* = 90°), *f*_c_ reaches the maximum value, which is equal to *f*_z_. In the conventional milling process, as shown in Figure 1c, the cutting section is approximately rectangular due to the relatively large axial depth of cut, *a*_p_. The uncut chip thickness is mainly determined by the feed per tooth *f*_z_. While in the precision milling process, the characteristics of the milling area are different from that in conventional milling. As shown in Figure 1d, when the milling parameter especially *a*_p_ is reduced to the micron level, which may be smaller than the tool corner radius *r*_ε_, the part of the cutting edge involved in cutting process is mainly located at the bottom of the tool nose. The cutting cross-section has an irregular shape whose thickness is gradually reduced from the surface to be machined to the surface that has been machined. Thus, there is a maximum uncut chip thickness, *h*_max_, as shown in Figure 2a. As shown in Figure 2b–d, the actual uncut chip thickness is different at different locations such as B1, B2, and B3 in the cutting zone.

According to the assumption of Befano [12], to get high machined surface quality, the uncut chip thickness *h* should be smaller than the critical chip thickness of materials *d*_c_, the material will be removed in ductile mode, and without brittle failure. A conservative parameter setting strategy is to keep the *h*_max_ less than the critical value *d*_c_ of the material [24]. Therefore, it could ensure the material will be removed in completely ductile mode without brittle failure. 

For the specific selected *f*_z_ and *a*_p_, and end mill with tool corner radius *r*_ε_, the maximum uncut chip thickness, *h*_max_, can be approximately calculated by the Equation (1), when $\sqrt{2Ra_{p}-a_{p}{}^{2}}>f_{z}$ [35].

(1) $$h_{\max}=r_{\varepsilon }-\sqrt{r_{\varepsilon }{}^{2}+f_{z}{}^{2}-2f_{z}\sqrt{2r_{\varepsilon }a_{p}-a_{p}{}^{2}}}.$$

It can be seen from the Equation (1) that the maximum uncut chip thickness *h*_max_ in the cutting zone is affected by the feed per tooth *f*_z_, axial depth of cut *a*_p_ and the corner radius of the tool nose *r*_ε_. Understanding the relationship between *h*_max_ and each cutting parameter helps guide the selection of cutting parameters. 

**Figure 3:**
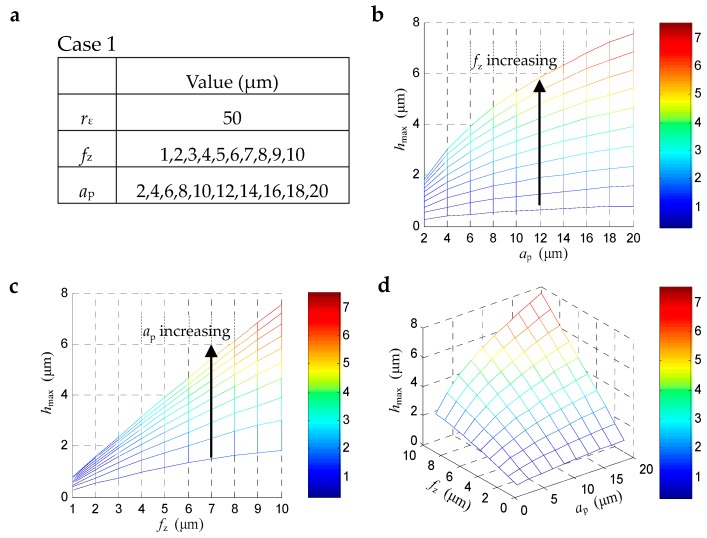
The maximum uncut chip thickness *h*_max_ changes with *f*_z_ and *a*_p_: (**a**) parameters setting, (**b**) 2D-view from the perspective of *a*_p_, (**c**) 2D-view from the perspective of *f*_z_, (**d**) 3D-view from both *f*_z_ and *a*_p_.

Figure 3 shows the maximum uncut chip thickness *h*_max_ changes with *f*_z_ and *a*_p_. For a specific end mill with tool corner radius *r*_ε_ about 50 μm, when the *f*_z_ changes from 1 to 10 μm, the *a*_p_ changes from 2 to 20 μm, the calculated *h*_max_ under each set of parameters show growing trend with the increasing of *f*_z_ and *a*_p_. The change of *f*_z_ has a greater impact on *h*_max_ than that of *a*_p_. It means for a specific cutting tool, smaller *f*_z_ will bring smaller *h*_max_.

**Figure 4:**
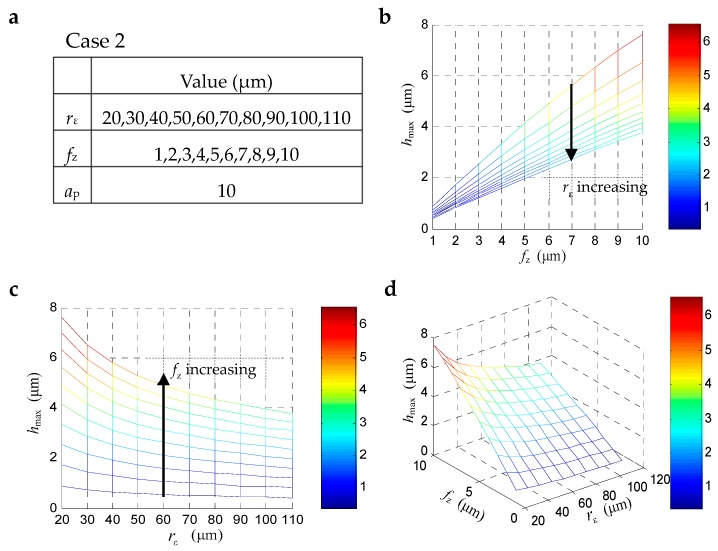
The maximum uncut chip thickness *h*_max_ changes with *f*_z_ and *r***_ε._:** (**a**) parameters setting, (**b**) 2D-view from the perspective of *f*_z_, (**c**) 2D-view from the perspective of *r***_ε_**, (**d**) 3D-view from both *f*_z_ and *r***_ε_**.

Figure 4 shows the maximum uncut chip thickness *h*_max_ changes with *r*_ε_. and *f*_z_. In this case, when the axial depth of cut *a*_p_ is a fixed on 10 μm, the *f*_z_ changes from 1 to 10 μm, and the tool corner radius *r*_ε_ changes from 20 to 100 μm, the *h*_max_ show different trends. It can be seen that the *h*_max_ increased with the increasing of *f*_z_ as usual, while decreased with the increasing of tool corner radius. It means that when machining with the given *f*_z_ and *a*_p_, cutting tools with a bigger corner radius will bring a smaller *h*_max_.

### 2.2. Parameters Selection Criteria in Precision Milling Ceramics

Generally, when the milling parameters are selected, as long as the maximum cutting thickness is limited to the critical thickness (*d*_c_) of the material brittle-plastic transition (*h*_max_ < *d*_c_), any position of the cutting arc region can be ensured that the uncut chip thickness is less than *d*_c_, and the machined surface for ductility removal can be obtained.

**Figure 5:**
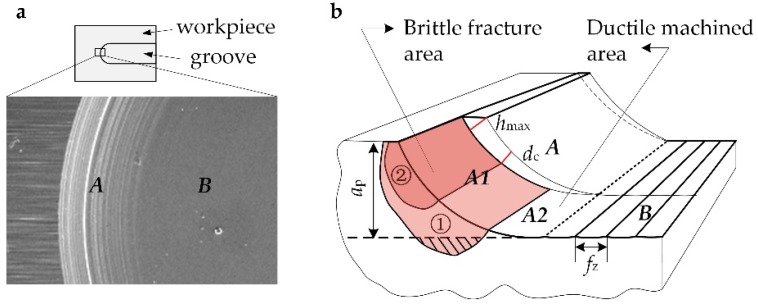
Schematic sketch of machined surface on groove edge: (**a**) different surface morphology of area A and B; (**b**) illustration of the effect of the brittle failure zone on the machined surface.

Figure 5 is a schematic sketch of the machined surface on the groove edge. During each cutting process, the material was removed by the cutting edge, and the machined surface area *A* was formed due to the tool corner radius. As the cutting process continues in the feed direction, the continuously generated new machined surface area *A* overlaps each other closely in accordance with the pitch of feed per tooth, *f*_z_, to form the machined surface area B (Figure 5a). Due to the existence of the tool corner radius, the closer to the machined surface *B* is, the smaller the uncut chip thickness is. Therefore, it is generally always machined in ductile mode near the machined surface area *B*.

According to the assumption of Befano [12], it can be deduced that when the *h*_max_ is bigger than the *d*_c_ of material, the material on corresponding position will occur brittle damage (marked with *A1*), as shown in Figure 5b. Additionally, the area *A2* where near the machined surface still show a smooth surface. If the brittle fracture area is large enough to extend below the machined surface, as the area marked with ①, the damaged area will not be removed completely during the subsequent cutting process. Part of the damaged area will finally remain on the machined surface area *B* and present a brittle fracture surface morphology. However, if the brittle fracture area is controlled in a small range, and does not extend to the machined surface, as the area marked with ②, these damaged areas will be removed during the following cutting process completely. Therefore, a smooth surface still can be obtained finally. This reveals that when *h*_max_ is appropriately larger than *d*_c_, the ductile surface can be still theoretically obtained. Therefore, when precision milling hard and brittle materials, the strategy of setting the milling parameters can be improved by appropriately increasing the *h*_max_, actually the *f*_z_ and *a*_p_, which will increase the material removal rate, and still have a chance to get asmooth machined surface.

## 3. Experimental Setups

### 3.1. Workpiece Material

As described above, ZrO_2_ ceramics is currently used in a broad range of industrial applications. The workpiece material employed in this work was an yttria-stabilized tetragonal zirconia (Y-TZP) with a 2 wt% of Al_2_O_3_. The starting powder mixture (TM2 grade) contained yttria-free monoclinic ZrO_2_ powder mixed in such a ratio that the overall content of Y_2_O_3_ stabilizer was 2 mol%. The material was prepared by hot pressing at a pressure of 28 MPa and at a temperature of 1450 °C for 1 h. The material was characterized by relative high fracture toughness and modest hardness; Table 1 summarizes relevant properties of the ZrO_2_ ceramic work piece used during investigation. 

### 3.2. PCD Micro End Mills

**Figure 6:**
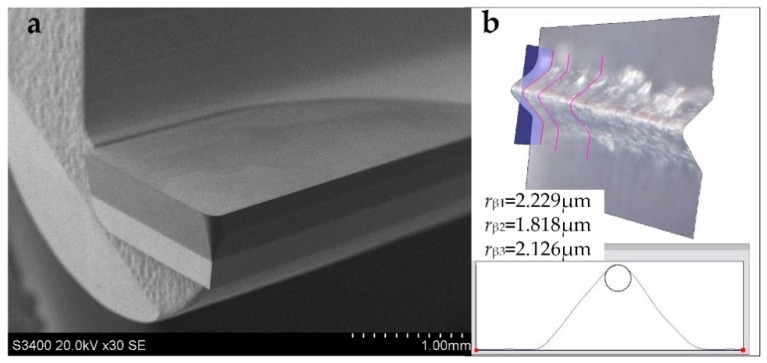
(**a**)SEM image of the polycrystalline diamond (PCD) end mill and (**b**) measuring of cutting edge radius.

The milling tools used in this study were single flute PCD end mills with a corner radius of 0.1 mm. The tool blank was made of a tungsten carbide tool shank and the PCD compact (Element 6, CMX850, average grain size *s* <1 μm) which was brazed on it. Figure 6 shows the structure and tool geometry of the PCD end mill. The cutting edge was designed to be straight in shape. The tool diameter was about 4 mm. The rake angle (α) and flank angle (γ) of the cutting edge were 0° and 5° respectively; hence, providing a robust cutting edge geometry to withstand cutting loads. The geometry of cutting edge was preformed by Wire cut Electrical Discharge Machining (WEDM), and then ground by a precision tool grinder. The cutting edge radius was measured to be less than 3 μm by a Leica DVM5000 microscope. The quality of the cutting edge was checked by scanning electron microscopy (SEM). The set of tool parameter is listed in Table 2.

### 3.3. Experimental Conditions and Procedures

In this study, two experimental campaigns were carried out. In order to clarify the characteristics of the machined surface when using corner radius end mill, the first experimental case 1 was set with fixed axial depth of cut, spindle rotating speed, but different feed per tooth, which resulted in different *h*_max_, as listed in case 1 in Table 3. After the experiments, the characteristics of the groove shoulder were observed by SEM to help understanding the material removal mechanism.

According to the previous analysis, when the end mill is selected, *h*_max_ is mainly determined by *f*_z_ and *a*_p_. The change of *f*_z_ has a greater influence on the value of *h*_max_. In the second part, case 2, by selecting different combinations of *f*_z_ and *a*_p_, the calculated *h*_max_ of each group can be set to am almost similar level about 2 μm. Therefore, from test 1 to 4, *f*_z_ appears to decrease gradually from 5 to 2 μm, and *a*_p_ exhibits a multiple increase from 10 μm to a relatively bigger value about 80 μm, as listed in case 2 in Table 3. Thus, the maximum uncut chip thickness *h*_max_ was limited in a small value, but the material removal rate was increased. After the experiments, the cutting force and machined surface roughness were analyzed to see the performance of using exploratory milling parameters.

**Figure 7:**
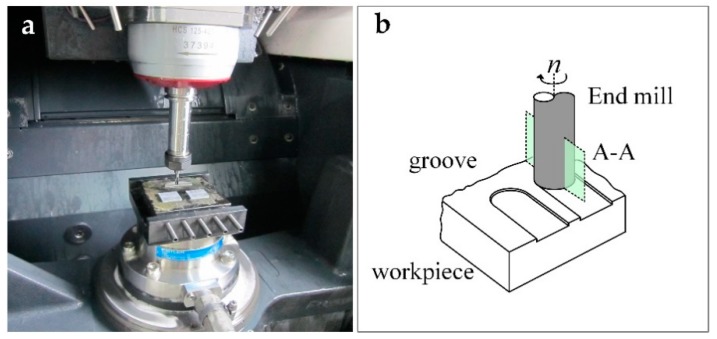
(**a**) Experimental setups and (**b**) a sketch of groove milling.

The experiments were conducted on a precision milling machine center (DMG Ultrasonic 20 linear, Stuttgart, Germany). A view of the experiment setups and the sketch of groove milling are shown in Figure 7. Workpieces were attached on the fixture with wax, and the surfaces of the samples were precisely ground to insure flatness and alignment. The fixture was then mounted on a dynamometer (Kistler type 9272, Kistler Group, Winterthur, Switzerland) used to measure the cutting force. The milling process was conducted in wet condition, using a water based emulsion to remove chips and debris. After the tests, the machined surface was cleaned by an ultrasonic cleaning machine and the surface roughness of the grooves were measured by a Mahr Perthomometer M1 (ISO 4287). The machined surface topography was inspected by an SEM. 

## 4. Results and Discussion

### 4.1. Possibility of Increasing Machining Efficiency

**Figure 8:**
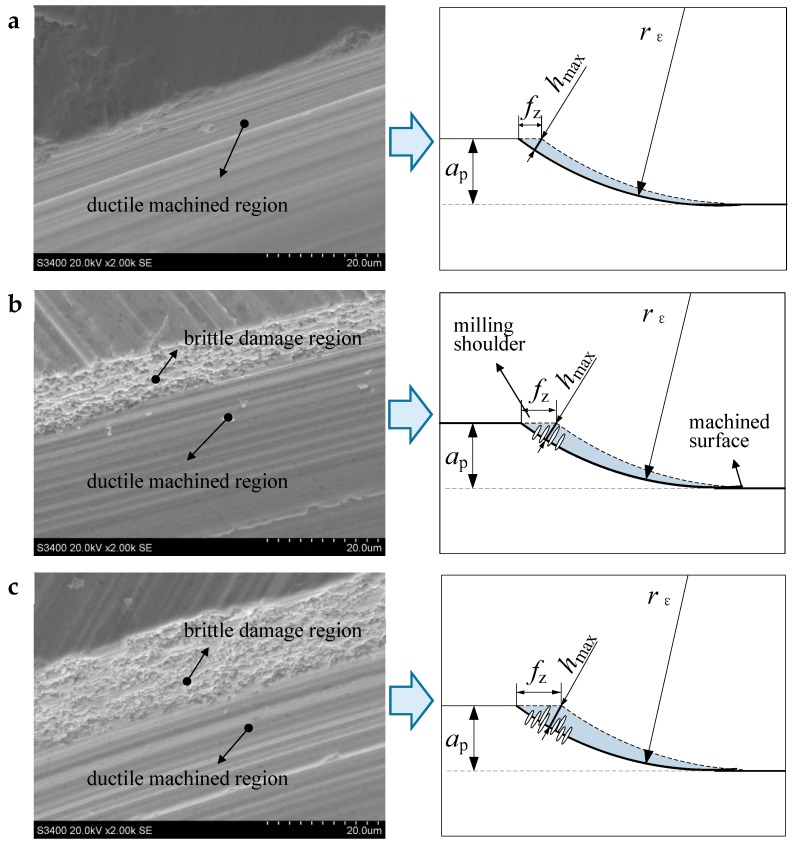
Characteristics of machined surface under different *h*_max_, (**a**) *f*_z_ = 3 μm, *a*_p_ = 20 μm, *h*_max_ = 1.8 μm (**b**) *f*_z_ = 8 μm, *a*_p_ = 20 μm, *h*_max_ = 4.5 μm (**c**) *f*_z_ = 10 μm, *a*_p_ = 20 μm, *h*_max_ = 5.7 μm.

Figure 8 shows the SEM images of the milling shoulder in the experiment case 1. The *h*_max_ in the three trials is 1.8, 4.5 and 5.7 μm, respectively. According to Bifano’s empirical formula, the critical uncut chip thickness of the test ZrO_2_, *d*_c_, is calculated to be about 2.6 μm by Equation (2):

(2) $$d_{c}=0.15\left(\frac{E}{H}\right){\left(\frac{K_{IC}}{H}\right)}^{2},$$

where *K_IC_* is the fracture toughness, *H* is the hardness, and *E* is the elastic modulus (see Table 1) [12]. Thus, in test 1, the *h*_max_ is about 1.8 μm, less than the critical value. The material in cutting area should be removed totally in ductile mode without brittle damage. As shown in Figure 8a, the machined area in milling shoulder presents a complete plastic texture. In test 2, due to the increase of *f*_z_, the *h*_max_ is increased to about 4.5 μm that is bigger than the size of *d*_c_. As shown in Figure 8b, the machined area near the milling shoulder where marked with *h*_max_, shows some brittle damage. However, the machined area away from the milling shoulder still shows the shape of ductile cutting. The same phenomenon also appears in test 3, as shown in Figure 8c, the brittle damage region is obviously larger than that in test 2. The results verify the previous analysis in part 2. When the maximum uncut chip thickness *h*_max_ is appropriately larger than *d*_c_, a certain degree of brittle failure occurs near the milled shoulder. When the damaged areas are limited, the machined surface could still exhibit a ductile removal morphology.

**Figure 9:**
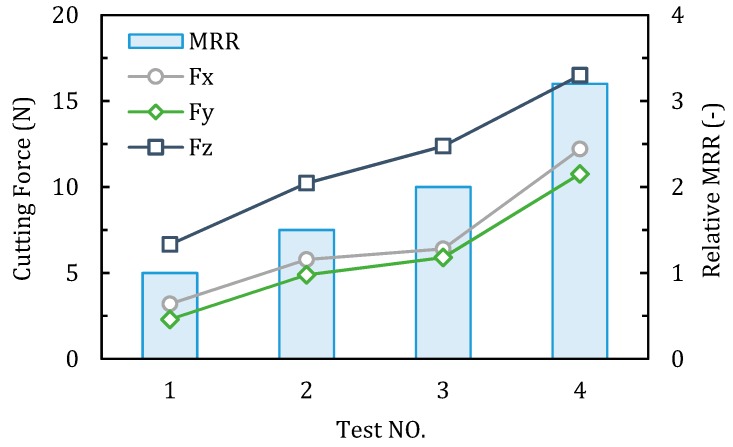
Cutting force and relative material removal rate (MRR) of each test in case 2.

**Figure 10:**
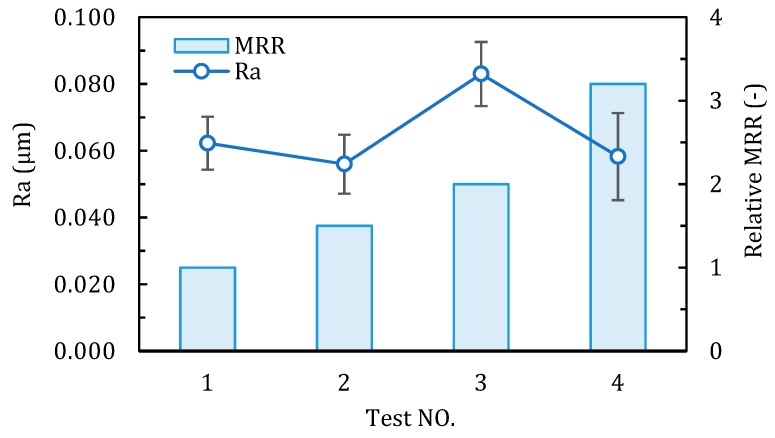
Surface roughness Ra and relative MRR of each test in case 2.

Figure 9 and Figure 10 show the comparison of milling force, the machined surface roughness Ra and the relative material removal rate (take the MRR of test 1 as one unit) of each test in case 2. From test 1 to 4, the feed per tooth (*f*_z_) was reduced by 60%, however, a significant increase of axial depth of cut (*a*_p_) increased the material removal rate by 2.2 times. As shown in Figure 9, the milling force presents an obvious increasing trend from test 1 to 4, due to the increasing of removal material volume. In each test, due to the existence of tool corner radius, the axial milling force Fz is much bigger than Fx and Fy, which can produce large compressive stress in the chip formation zone, and it is reported benfitial for the ductile machining process [36]. It can be seen from the Figure 10, the measured surface roughness Ra of the four tests are all below 0.1 μm. Specifically, the Ra in test 3 is the largest with the average value about 0.083 μm, and in the other three tests are around 0.06 μm. It is revealed that when machining ceramics by corner radius end mill, the proper increase in the axial depth of cut can improve the material removal rate, while still possible to achieve a satisfactory surface roughness.

### 4.2. Material Removal Mechanism

**Figure 11:**
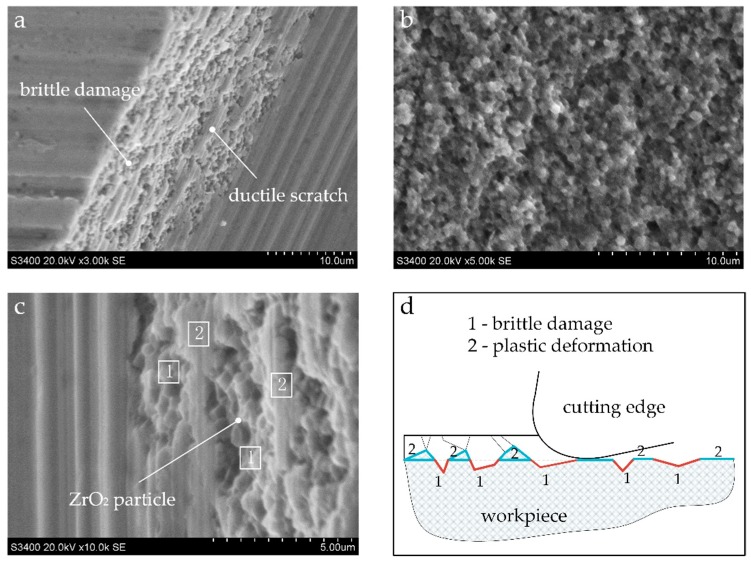
SEM image of (**a**) brittle fracture and plastic deformation in groove edge; (**b**) natural fracture surface of zirconia; (**c**) subtle view of micro damaged area and plastic scratched area; (**d**) schematic sketch of the material removal mechanism.

Figure 11 shows the topography of the brittle fracture in the milled shoulder and a schematic sketch to help understand the material removal mechanism. In the area near milling shoulder, the actual uncut chip thickness is greater than the critical uncut chip thickness of the material brittle plastic transition. The material removal mode is mainly based on local brittle fracture, however, some scratches in the plastic deformation mode also can be found to be distributed in the brittle failure zone, as shown in Figure 11a. The overall morphology is somewhat different from the natural fracture surface morphology of zirconia that exhibits an uneven, rough, and highly grainy appearance (see Figure 11b). A more subtle view in Figure 11c shows that regular zirconia particles are visible in the brittle failure zone where always presented in the form of pits marked with a 1. The plastic scratched area (marked with a 2 in Figure 11c) exhibits a smooth surface.

As can be understood from Figure 11d, during the cutting process, the material to be cut is subjected to brittle fracture, and the crack is generated and expanded in the front to cause brittle fracture of the material to form an irregular fractured surface. When the cutting edge is swept, the material below the cutting surface (marked with a 1) does not contact the cutting edge and forms a lot of pits that present the original fractured surface morphology, and the material above the cutting surface are plastically deformed due to the pressing and ploughing action of the cutting edge during the cutting process. The zirconia particles are finally plastically deformed into a smooth micro plane (marked with a 2) with ductile scratches. In general, from the microscopic characteristics, the material in the brittle fracture zone is broken along the crystal, and the material in plastic scratch zone is removed with plastic deformation in the manner of transgranular failure. It was also found in the test of grinding zirconia ceramics with diamond tools by Mohammad [37].

## 5. Conclusions

This paper presents a study on the performance of corner radius end mill in precision milling of zirconia ceramics with exploratory cutting parameters. Characteristics of precision milling ceramics and parameters selection criteria were discussed. Based on the results, the following conclusions have been drawn:

- When precision milling ZrO_2_ ceramics with a specific end mill, the change in the feed per tooth *f*_z_ has a greater influence on the maximum uncut chip thickness *h*_max_ than that in axial depth of cut *a*_p_. When *f*_z_ and *a*_p_ are constant, increasing the corner radius of the end mill can reduce the calculated *h*_max_.
- It is advisable to increase the feed per tooth appropriately during precision milling ZrO_2_ ceramics with corner radius end mills. When the calculated *h*_max_ is bigger than the critical value, even if the milling shoulder has brittle fractured, there is still a chance to obtain a ductile processed surface, as long as the brittle failure area is controlled within a certain range. Appropriate increase of the axial depth of cut, *a*_p_, not only can prevent the minor brittle damage from affecting the machined surface, but also could increase the material removal rate. The milling force increases with increasing of machining efficiency, but the surface roughness can still be stabilized within a certain range.
- In the area near the milling shoulder, the actual uncut chip thickness is greater than the critical uncut chip thickness of the material brittle plastic transition. The machined surface morphology is mainly based on local brittle fractured pits and accompanied by some ductile scratches caused by plastic deformation. The material in brittle fracture zone is broken along the crystal, and the material in the plastic scratch zone is removed with plastic deformation in the manner of transgranular failure.

This study is an initial work on high performance milling of engineering ceramics. It gives a direction on how to improve the milling efficiency from the perspective of selection milling parameters. As for the extent to which the cutting parameters can be increased, further research is needed.

## Figures and Tables

**Table 1:** Chemical composition and mechanical properties of the workpiece material.

**Composition**	
Y_2_O_3_ content (mol%)	2
Al_2_O_3_ content (wt%)	2
**Physical and Mechanical Properties**	
Density *ρ* (g/cm^3^)	6.02
Young’s modulus *E* (GPa)	223
Fracture Toughness *K_IC_* (MPam^1/2^)	11.1 ± 0.7
Hardness HV_10_ (kg/mm^2^)	1180 ± 13

**Table 2:** Tool parameters of the PCD corner radius end mills.

Tool Parameters	Value
grain size *s* (μm)	< 1
Tool diameter *D* (mm)	4
Tool corner radius *r*_ε_ (mm)	0.1
Rake angle *α* (°)	0
Flank angle *γ* (°)	5
Cutting edge radius *r*_β_ (μm)	< 3

**Table 3:** Experimental parameters of the two cases.

No.	Spindle Rotating Speed *n*/rpm	Feed Per Tooth *f*_z_/μm	Milling Depth *a*_p_/μm	Maximum Uncut Chip Thickness *h*_max_/μm *
Case 1				
1	8000	3	20	1.8
2		8	20	4.5
3		10	20	5.7
Case 2				
1	8000	5	10	2.08
2		3	25	1.96
3		2.5	40	1.99
4		2	80	1.96

* Tool corner radius *r*_ε_ 0.1 mm.

## References

[1] Liu K., Reynaerts D., Lauwers B. (2009). Influence of the pulse shape on the edm performance of si3n4–tin ceramic composite. CIRP Ann..

[2] Barry C.C., Grant N.M. (2007). Ceramic Materials/Science and Engineering.

[3] Ferraris E., Vleugels J., Guo Y., Bourell D., Kruth J.P., Lauwers B. (2016). Shaping of engineering ceramics by electro, chemical and physical processes. CIRP Ann..

[4] Denry I., Holloway J. (2010). Ceramics for dental applications: A review. Materials.

[5] Fernández-Valdivielso A., López de Lacalle L., Urbikain G., Rodriguez A. (2016). Detecting the key geometrical features and grades of carbide inserts for the turning of nickel-based alloys concerning surface integrity. Proc. Inst. Mech. Eng. Part C J. Mech. Eng. Sci..

[6] Urbikain G., de Lacalle L.N.L. (2018). Modelling of surface roughness in inclined milling operations with circle-segment end mills. Simul. Model. Pract. Theory.

[7] Ghani A.K., Choudhury I.A., Husni (2002). Study of tool life, surface roughness and vibration in machining nodular cast iron with ceramic tool. J. Mater. Process. Technol..

[8] Urbikain G., López de Lacalle L.N., Fernández A. (2014). Regenerative vibration avoidance due to tool tangential dynamics in interrupted turning operations. J. Sound. Vib..

[9] Polvorosa R., Suárez A., de Lacalle L.N.L., Cerrillo I., Wretland A., Veiga F. (2017). Tool wear on nickel alloys with different coolant pressures: Comparison of alloy 718 and waspaloy. J. Manuf. Process..

[10] Urbikain G., Artetxe E., López de Lacalle L.N. (2017). Numerical simulation of milling forces with barrel-shaped tools considering runout and tool inclination angles. Appl. Math. Model..

[11] Shimada S., Ikawa N., Inamura T., Takezawa N., Ohmori H., Sata T. (1995). Brittle-ductile transition phenomena in microindentation and micromachining. CIRP Ann. Manuf. Technol..

[12] Bifano T.G., Dow T.A., Scattergood R.O. (1991). Ductile-regime grinding—A new technology for machining brittle materials. J. Eng. Ind..

[13] Beltrão P.A., Gee A.E., Corbett J., Whatmore R.W. (1999). Ductile mode machining of commercial pzt ceramics. CIRP Ann. Manuf. Technol..

[14] Zhong Z.W. (2003). Ductile or partial ductile mode machining of brittle materials. Int. J. Adv. Manuf. Technol..

[15] Yanyan Y., Bo Z., Junli L. (2009). Ultraprecision surface finishing of nano-zro2 ceramics using two-dimensional ultrasonic assisted grinding. Int. J. Adv. Manuf. Technol..

[16] Ferraris E., Reynaerts D., Lauwers B. (2011). Micro-edm process investigation and comparison performance of al3o2 and zro2 based ceramic composites. CIRP Ann. Manuf. Technol..

[17] Liu K., Ferraris E., Peirs J., Lauwers B., Reynaerts D. (2008). Micro-edm process investigation of si3n4–tin ceramic composites for the development of micro fuel-based power units. Int. J. Manuf. Res. (IJMR).

[18] Shahzad K., Deckers J., Boury S., Neirinck B., Kruth J.P., Vleugels J. (2012). Preparation and indirect selective laser sintering of alumina/pa micro spheres. Ceram. Int..

[19] Shahzad K., Deckers J., Zhang Z., Kruth J.P., Vleugels J. (2014). Additive manufacturing of zirconia parts by indirect selective laser sintering. J. Eur. Ceram. Soc..

[20] Scheithauer U., Weingarten S., Johne R., Schwarzer E., Abel J., Richter H.-J., Moritz T., Michaelis A. (2017). Ceramic-based 4d components: Additive manufacturing (am) of ceramic-based functionally graded materials (fgm) by thermoplastic 3d printing (t3dp). Materials.

[21] Ehmann K.F., Devor R.E., Kapoor S.G. (2002). Micro/meso-scale mechanical manufacturing–opportunities and challenges. JSME/ASME Int. Conf. Mater. Process..

[22] Dhanorker A., Ozel T. (2008). Meso/micro scale milling for micro-manufacturing. Int. J. Mech. Manuf. Syst..

[23] Dornfeld D., Min S., Takeuchi Y. (2006). Recent advances in mechanical micromachining. CIRP Ann. Manuf. Technol..

[24] Bian R., Ferraris E., He N., Reynaerts D. (2014). Process investigation on meso-scale hard milling of zro2 by diamond coated tools. Precis. Eng..

[25] Bian R., He N., Ding W., Liu S. (2017). A study on the tool wear of pcd micro end mills in ductile milling of zro2 ceramics. Int. J. Adv. Manuf. Technol..

[26] Arif M., Rahman M., San W.Y. (2013). A study on the effect of tool-edge radius on critical machining characteristics in ultra-precision milling of tungsten carbide. Int. J. Adv. Manuf. Technol..

[27] Wu X., Li L., He N., Zhao G., Jiang F., Shen J. (2018). Study on the tool wear and its effect of pcd tool in micro milling of tungsten carbide. Int. J. Refract. Met. Hard Mater..

[28] Bai J., Bai Q., Tong Z. (2017). Multiscale analyses of surface failure mechanism of single-crystal silicon during micro-milling process. Materials.

[29] Zhong L., Li L., Wu X., He N. (2017). Micro cutting of pure tungsten using self-developed polycrystalline diamond slotting tools. Int. J. Adv. Manuf. Technol..

[30] Matsumura T., Ono T. (2008). Cutting process of glass with inclined ball end mill. J. Mater. Process. Technol..

[31] Cheng X., Nakamoto K., Sugai M., Matsumoto S., Wang Z.G., Yamazaki K. (2008). Development of ultra-precision machining system with unique wire edm tool fabrication system for micro/nano-machining. CIRP Ann. Manuf. Technol..

[32] Nakamoto K., Katahira K., Ohmori H., Yamazaki K., Aoyama T. (2012). A study on the quality of micro-machined surfaces on tungsten carbide generated by pcd micro end-milling. CIRP Ann. Manuf. Technol..

[33] Zhan Z., He N., Li L., Shrestha R., Liu J., Wang S. (2014). Precision milling of tungsten carbide with micro pcd milling tool. Int. J. Adv. Manuf. Technol..

[34] Bian R., Ferraris E., Ynag Y., Qian J. (2018). Experimental investigation on ductile mode micro-milling of zro2 ceramics with diamond-coated end mills. Micromachines.

[35] Liu K., Li X., Rahman M., Neo K., Liu X. (2007). A study of the effect of tool cutting edge radius on ductile cutting of silicon wafers. Int. J. Adv. Manuf. Technol..

[36] Liu K., Li X., Liang S. (2007). The mechanism of ductile chip formation in cutting of brittle materials. Int. J. Adv. Manuf. Technol..

[37] Rabiey M., Jochum N., Kuster F. (2013). High performance grinding of zirconium oxide (zro2) using hybrid bond diamond tools. CIRP Ann. Manuf. Technol..

